# Accuracy of Lung Ultrasonography versus Chest Radiography for the Diagnosis of Adult Community-Acquired Pneumonia: Review of the Literature and Meta-Analysis

**DOI:** 10.1371/journal.pone.0130066

**Published:** 2015-06-24

**Authors:** Xiong Ye, Hui Xiao, Bo Chen, SuiYang Zhang

**Affiliations:** 1 Department of Respiratory Medicine, Shanghai Pudong Hospital/Fudan University Pudong Medical Center, Shanghai, China; 2 Department of Respiratory Medicine, Shanghai Jiaotong University affiliated Shanghai First People’s Hospital, Shanghai, China; 3 Department of Ultrasound Medicine, the Second Artillery General Hospital, Beijing, China; 4 Department of Respiratory Medicine, the Second Artillery General Hospital, Beijing, China; University of Dundee, UNITED KINGDOM

## Abstract

Lung ultrasonography (LUS) is being increasingly utilized in emergency and critical settings. We performed a systematic review of the current literature to compare the accuracy of LUS and chest radiography (CR) for the diagnosis of adult community-acquired pneumonia (CAP). We searched in Pub Med, EMBASE dealing with both LUS and CR for diagnosis of adult CAP, and conducted a meta-analysis to evaluate the diagnostic accuracy of LUS in comparison with CR. The diagnostic standard that the index test compared was the hospital discharge diagnosis or the result of chest computed tomography scan as a “gold standard”. We calculated pooled sensitivity and specificity using the Mantel-Haenszel method and pooled diagnostic odds ratio using the DerSimonian-Laird method. Five articles met our inclusion criteria and were included in the final analysis. Using hospital discharge diagnosis as reference, LUS had a pooled sensitivity of 0.95 (0.93-0.97) and a specificity of 0.90 (0.86 to 0.94), CR had a pooled sensitivity of 0.77 (0.73 to 0.80) and a specificity of 0.91 (0.87 to 0.94). LUS and CR compared with computed tomography scan in 138 patients in total, the Z statistic of the two summary receiver operating characteristic was 3.093 (P = 0.002), the areas under the curve for LUS and CR were 0.901 and 0.590, respectively. Our study indicates that LUS can help to diagnosis adult CAP by clinicians and the accuracy was better compared with CR using chest computed tomography scan as the gold standard.

## Introduction

Community-acquired pneumonia (CAP) is a major health problem worldwide, failure of early detection and distribution of treatment may lead to significant morbidity and mortality [1]. International guidelines recommend the use of chest radiography (CR) as routine evaluation of a patient suspected of pneumonia, but CR has been demonstrated to be an insensitive method with relatively low accuracy[2,3]. Wesley H. S and his colleagues evaluated the use of CR with computed tomography (CT) scanning for detection of pulmonary opacities[4]. In 3,423 adult of emergency department patients with acute cardiopulmonary symptoms, the sensitivity of CR was 43.5% (95% CI, 36.4%-50.8%) and the positive predictive value was 26.9% (95% CI, 22.1%-32.2%) of the final radiologist reports of noted opacity, infiltrate, consolidation, pneumonia, or bronchopneumonia. Thoracic CT scan is considered the “gold standard” for detection of pneumonia and other pulmonary lesions, but it cannot be used as a first-line radiological examination in all patients with suspected pneumonia. This is mainly due to the fact that it is often costly, not available and that it involves a high radiation dose [5].

The interest in lung ultrasounds (LUS) has increased during the last few years for the use in diagnosis of pleural effusions, pneumothorax, pneumonia, pulmonary embolism and pulmonary contusions [6–9]. We undertook this meta-analysis of the published literatures to compare the accuracy of LUS and CR in the diagnosis of adult CAP.

## Materials and Methods

### Study Design and Data Sources

We systematically reviewed the literature of published research articles evaluating the diagnostic accuracy of LUS in comparison with CR. Original articles in English performed in adult populations published up to the end of May 2014 were searched in Pub Med and EMBASE databases. We used combinations of the following key words to identify all original studies in which ultrasonography and CR were used in diagnosing clinically suspected CAP (“ultrasound” or “sonography” or “ultrasonography” or “radiography” or “chest film” or “chest radiograph”) and (“pneumonia”). Articles that suggested to be related by Pub Med or EMBASE were also retrieved. Bibliographies of retrieved articles were searched independently and checked for additional studies.

### Study Selection

The inclusion criteria we used to select articles were as follows that similar to Alrajab S et al, they made a meta analysis of pleural ultrasonography versus CR for the diagnosis of pneumothorax [10]: (1) Original studies comparing the performance of LUS and CR for the detection of clinical suspected adult CAP with the following symptoms (cough, sputum production, fever, pleuritic chest pain and dyspnoea) and/or signs (rales or bronchial breath sounds), in accordance with American Thoracic Society guidelines[11]; (2) Comparison of a composite standard that the diagnosis of hospital discharge by physicians in charge of the patients on the basis of clinical evolution, CR, markers of inflammation and microbiology or imaging results with chest CT scan if available; (3) Reporting of results in sufficient detail to allow reconstruction of contingency tables of the raw data (true-positive, true-negative, false-positive and false-negative results); (4) Described the diagnostic criteria for pneumonia on LUS in clear details (i.e. the presence of an unilateral or a bilateral alveolar-interstitial syndrome, focal interstitial syndrome and the finding of subpleural lung consolidation with evidence of static or dynamic air bronchograms)[12]; (5) Meeting quality standards, as assessed by the 14-item Quality Assessment of Diagnostic Accuracy Studies (QUADAS) tool[13].

### Review process

**Fig 1:**
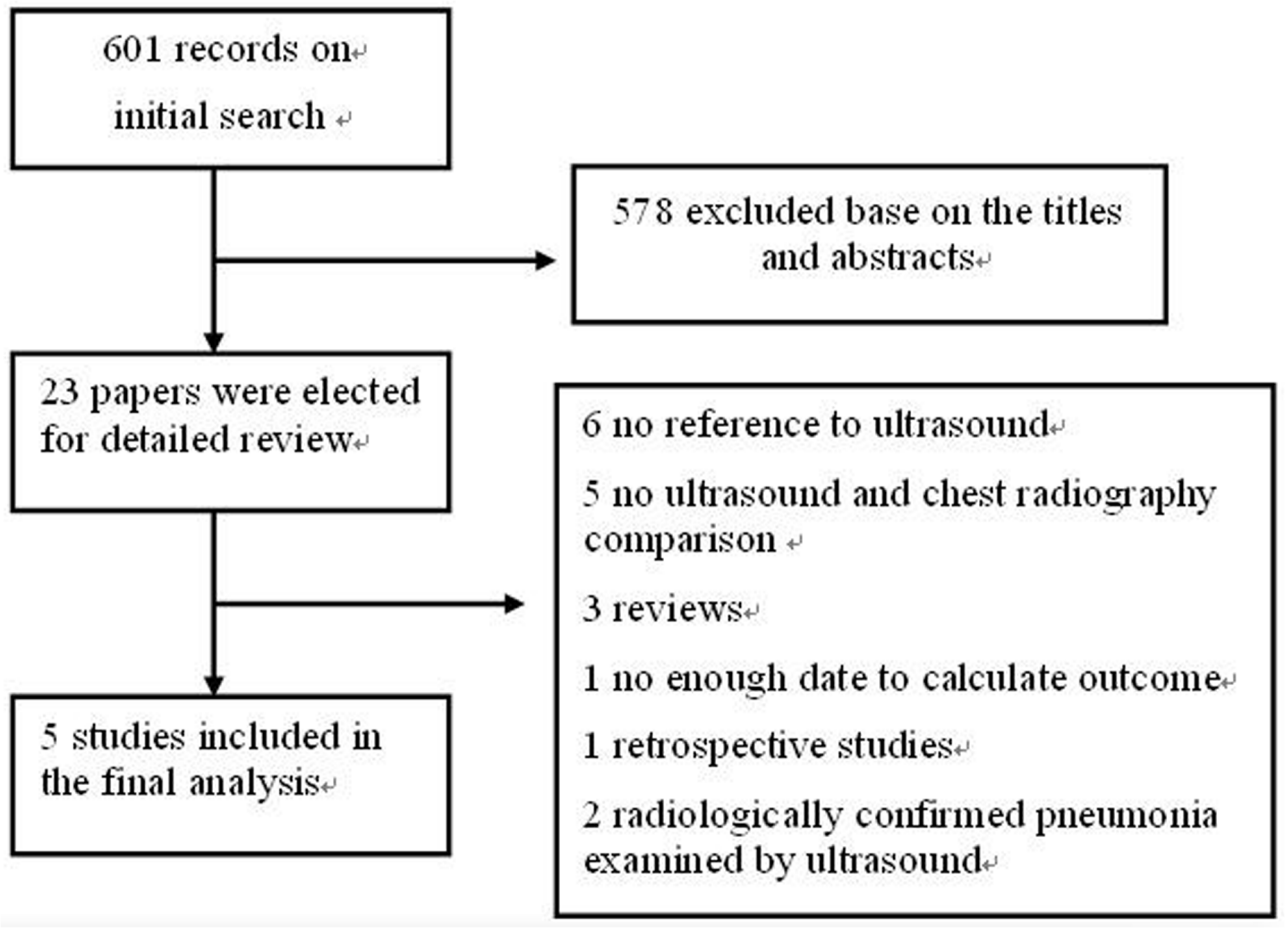
Flow chart for study selection.

Two authors (X.Y and H.X) independently reviewed the articles and ascertained the criteria for inclusion in the pooled data analysis, with disagreements resolved by discussion. We excluded two studies with radiologically confirmed pneumonia examined by ultrasound [14,15], due to the inclusion of known diseased population in meta-analyses of diagnostic tests will overestimate the diagnostic odds ratio (DOR) by increasing the odds of having a positive test in diseased subjects[16]. Characteristics of the included and excluded articles are presented in Fig 1.

### Data Analysis

We used the Meta-DiSc, version 1.4 software (Ramon y Cajal Hospital, Madrid, Spain) in our meta-analysis. The Mantel-Haenszel method of the random-effect model was used to calculate pooled sensitivity and specificity with corresponding 95% confidence intervals, and pooled DOR using the DerSimonian-Laird method. The Spearman correlation coefficient between the logit of sensitivity and 1-specificity was calculated to test the threshold/cutoff effect. Other data such as summary receiver operating characteristic (sROC) curves were also obtained. Meta-DiSc computes the inconsistency index (I square) to quantify the effect of heterogeneity, which describes the percentage of total variation across studies that is due to heterogeneity rather than chance[17]. The statistical heterogeneity to be low for I ^2^ = 25 to 49%, moderate for I ^2^ = 50 to 74%, and high for I ^2^ >75%[17]. Some statistics implemented by Meta Disc cannot be calculated due to there were 0 values in some cells, we added 1/2 to all cells in all studies for correction [18]. We use the MedCalc Statistical Software version 13.0.2 (MedCalc Software bvba, Ostend, Belgium; http://www.medcalc.org) to calculate Z statistic of the sROC to compare the diagnostic accuracy of LUS and CR, with P <0.05 to have statistical significance.

## Results

**Fig 2:**
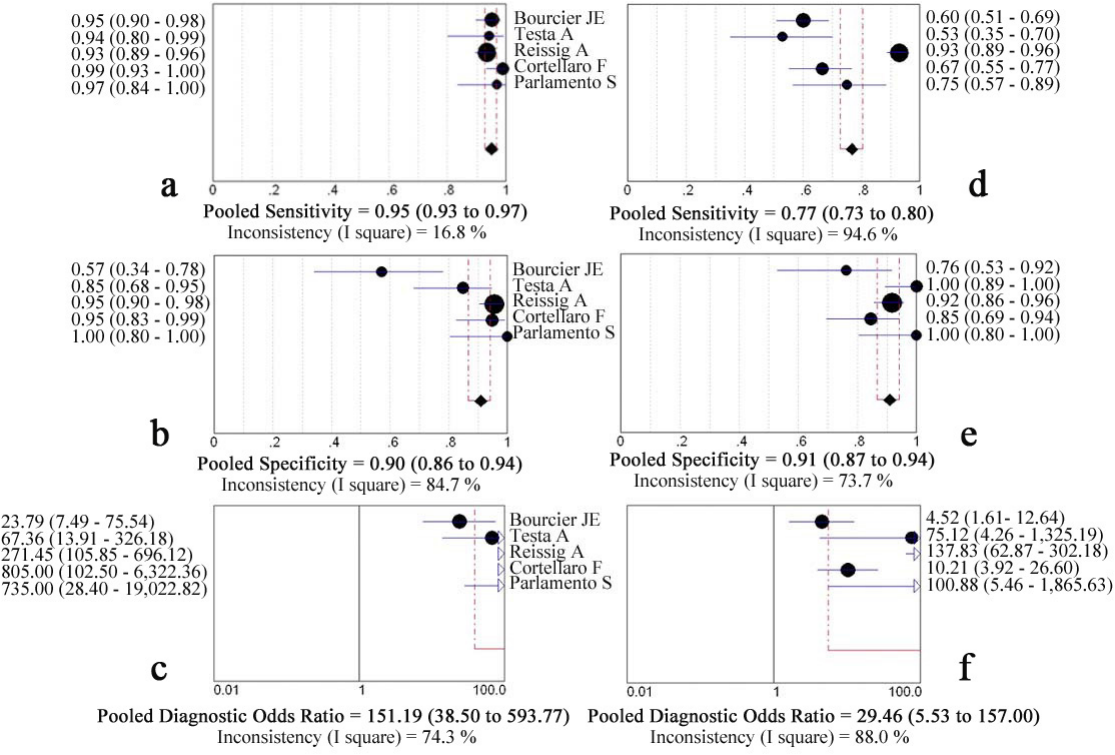
Forest plot for pooled sensitivity, specificity and diagnostic odds ratio of lung ultrasonography (a, b, c) and chest radiography (d, e, f) for the detection of pneumonia compared with hospital discharge diagnosis.

Five studies passed all inclusion criteria and were included in final analysis, showed in Table 1[19–23]. LUS was performed before CR by trained emergency physicians in all studies. Among the 742 patients in the five studies, chest CT scans were performed in 71 patients with equivocal or negative radiographic but positive LUS results in two studies [19,21], and in other three studies CT scans were obtained when considered clinically indicated or due to difficult diagnosis in 67 patients [20,22,23]. All of the included studies passed most of the fourteen QUADAS items, Table 2 summarizes the four informative questions, and the other ten which did not differ between studies were ‘positive’ answers that did not introduce significant bias. The feasibility of LUS was 100% without any discomfort in all subjects. Using hospital discharge diagnosis as reference standard, the calculated pooled sensitivity for LUS and CR were 0.95 (0.93–0.97) and 0.77 (0.73 to 0.80), respectively; the pooled specificity for LUS and CR were 0.90 (0.86 to 0.94) and 0.91 (0.87 to 0.94), respectively; pooled DOR for LUS was 151.19 (38.50 to 593.77), whereas for CR, the pooled DOR was 29.46 (5.53 to 157.00), Fig 2. The areas under the curve (AUC) for LUS and CR were 0.973 (SE, 0.014) and 0.912 (SE, 0.050), Z statistic compared with these two sROC was 1.365 (P = 0.172).

**Table 1:** The characteristics of included studies.

Study	Plans of	No.	Pre.		LUS				CR		
(Country)	CR		(%)	TP	FP	FN	TN	TP	FP	FN	TN
**Bourcier JE[23]**	Unreported	144	85.4	117	9	6	12	74	5	49	16
**(France)**				(95.1)			(57.1)	(60.2)			(76.2)
**Testa A [22]**	100% P	67	50.7	32	5	2	28	18	0	16	33
**(Italy)**				(94.1)			(84.8)	(52.9)			(100)
**Reissig A [21]**	100% P+L	362	63.3	214	6	15	127	213	11	16	122
**(Germany)**				(93.4)			(95.5)	(93)			(91.7)
**Cortellaro F[20]**	75%P	120	67.5	80	2	1	37	54	6	27	33
**(Italy)**	25%P+L			(98.8)			(94.9)	(66.7)			(84.6)
**Parlamento S [19]**	66%P	49	65.3	31	0	1	17	24	0	8	17
**(Italy)**	34%P+L			(96.9)			(100)	(75)			(100)

CR = chest radiography; FN = false negative; FP = false positive; LUS = lung ultrasonography; Pre. (%) = Prevalence of pneumonia. P = Poster-anterior; L = Lateral; P+L = Poster-anterior/Lateral; TN = true negative; TP = true positive.

**Table 2:** Details of quality assessment by the QUADAS tool.

Citation	Verification procedure biases	Interpretation biases		
	Did patients receive the same reference standard regardless of the index test result?	Were the index test results interpreted without knowledge of the results of the reference standard?	Were the reference standard results interpreted without knowledge of the results of the index test?	Were uninterpretable/ intermediate test results
**Bourcier JE**	No. 29/144 underwent a CT scan	Unreported	Unreported	NO
**Testa A**	No. 8/67 were performed CT scan	YES	YES	NO
**Reissig A**	No. 63/362 had low-dose CT scan	YES	YES	YES
**Cortellaro F**	No. 30/120 were performed CT scan	YES	YES	NO
**Parlamento S**	No. 8/49 were performed CT scan	YES	YES	NO

**Fig 3:**
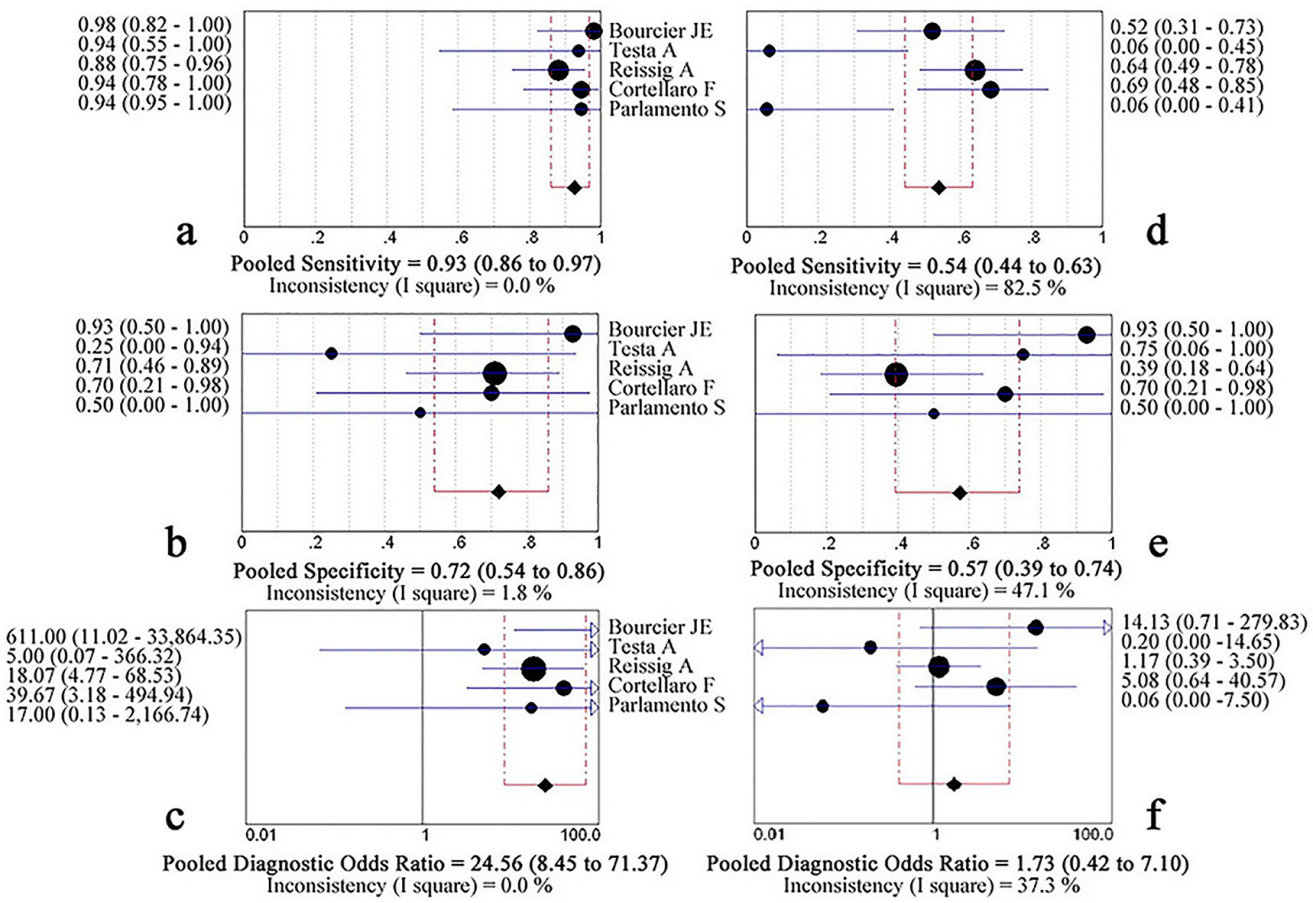
Forest plot for pooled sensitivity, specificity and diagnostic odds ratio of lung ultrasonography (a, b, c) and chest radiography (d, e, f) for the detection of pneumonia compared with chest computed tomography diagnosis.

**Fig 4:**
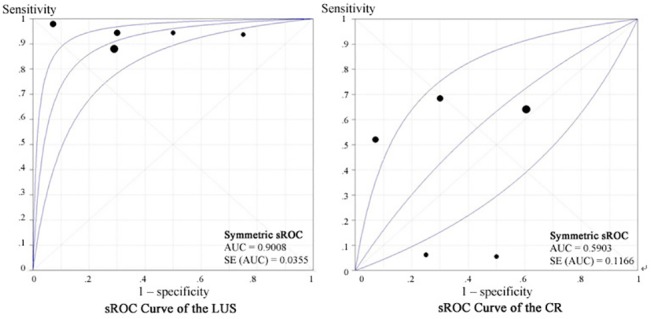
The summary receiver operating characteristic of lung ultrasonography and chest radiography for the detection of pneumonia compared with computed tomography scan. AUC: areas under the curve; CR: chest radiography; LUS: lung ultrasonography; sROC: summary receiver operating characteristic.

138 patients with conflicting results from LUS and CR were further studied with chest CT scan in all included studies. The LUS was 0.93 (0.86–0.97) sensitive and 0.72 (0.54–0.86) specific, with a pooled DOR of 24.56 (8.45 to 71.37) and an AUC of 0.901(SE, 0.036). In comparison, CR had a sensitivity of 0.54 (0.44–0.63), a specificity of 0.57 (0.39 to 0.74), a pooled DOR of 1.73 (0.42–7.10), and an AUC was 0.590(SE, 0.117), Figs 3 and 4. The Z statistic between LUS and CR of these two sROC curves was 3.093 (P = 0.002).

The Spearman correlation coefficient between the log of sensitivity and 1-specificity was -0.100(P = 0.873) for LUS and 0.100 (P = 0.873) for CR at hospital discharge diagnosis, and was -0.359 (P = 0.553) for LUS, 0.100 (P = 0.873) for CR at CT scans as a reference, these indicated that there were causes of variations other than a cutoff effect.

## Discussion

In this systematic review and meta-analysis, we found that the performance of LUS for detection of adult CAP was excellent, both in the hospital discharge diagnosis and in “gold standard” (chest CT scan). To our knowledge, this is the first time to compare LUS, CR and CT scan in the same patient for diagnosing of adult CAP. The accuracy of LUS was better than that of CR in 138 patients who further studied with a CT scan, however, there was no significant difference that used hospital discharge diagnosis as a reference by comparing the two sROC curves using Z statistic. The possible reason was that CR findings played a part in the hospital discharge diagnosis; there was false positive in CR to detect CAP in one study [21]. Heterogeneity of the results was expected a priori, and was accounted for using the random-effects meta-analysis framework. Some high I ^2^ shown in the forest plots, implied that there were significant heterogeneous across studies. First, the experience of the operator for LUS was inconsistency. The operation of ultrasound examination is strictly dependent on the experience of the operator, however, the image interpretation itself is definitely less dependent on the operator[24]. Second, CR was obtained in the supine or seated poster-anterior view only in some patients. Finally, Chest CT was performed in a limited number of non-randomized patients, so, more well designed randomized controlled trails focuses on detecting adult CAP using LUS comparison with CT scan is needed.

LUS performance is probably very good at detecting superficial pneumonia, it remains however poor at detecting deep alveolar lesions[25]. A CT scan of the chest is necessary with negative ultrasound results, in about 8% of the patients, CAP may not be detected by LUS [14]. Silva S et al investigated the clinical relevance of early general LUS in the ICU in patients with acute respiratory failure. The receiver operating characteristic curve analysis showed greater diagnostic performance of ultrasound in cases of 33 pneumonia patients compared with CR[26]. The use of cardiothoracic ultrasound appears to be an attractive complementary diagnostic tool and seems able to contribute to a rapid point-of-care triage and management of CAP patients. False-positive examinations were observed for LUS, mainly due to sepsis of other origin, pulmonary embolism, acute respiratory distress syndrome and pulmonary fibrosis. The number of cases with positive LUS and negative CR is sharply superior to the number of patients with negative LUS and positive CR. Fluid bronchogram reflects airways filled with fluid or secretions following airway obstruction, differential diagnosis of lung carcinoma should be taken into account in this case. Among one of the patients with fluid bronchogram in our included studies, a lung carcinoma was diagnosed 3 months later. Comorbidies such as congestive heart failure (initial pulmonary edema) and antibiotic therapy may also influence sonographic features[27]. The possibility of a dynamic evaluation gives ultrasound an advantage over CR, and possibly also over CT scan[28].

There were two published meta-analyses that conducted by Hu et al and Chavez, M. A et al evaluated the diagnostic accuracy of ultrasound for detecting pneumonia with very high sensitivity (97% and 94%) and specificity (94% and 96%) [29,30]. Hu et al included studies in children (n = 5) and Chavez, M. A et al included studies in critically ill patients (n = 4) whereas we limited our analysis to adults CAP. LUS perform better in children, which may help explain why Hu et al found a higher sensitivity than we did. In some include studies of Chavez M.A’s work did not assess all lung regions, as some patients were bedridden and their posterior zones were difficult to be assessed by LUS. Both of these two published articles did not compare the accuracy between LUS and chest X-ray in detection of CAP.

The present analysis has some limitations. We did not include articles in languages other than English, and did not try to identify studies that not be published in peer reviewed journals. The different qualification of the individuals performing the LUS and interpreting the CR in each of the studies may consider the potential source of bias. The small number of studies included in our meta-analysis is another limitation and we can’t perform meta-regression and subgroup analyses of the different lever of the operators’ experiences, as the accuracy of LUS and CR in the diagnosis of CAP depends on the skills of the operators.

## Conclusions

This study indicates that LUS can help recognize adult CAP. Using chest CT scan as a reference, the diagnostic accuracy of LUS better than that of CR in adult patients with clinically suspected community-acquired pneumonia.

## Supporting Information

S1 Checklist Checklist item. (DOC)
